# A study of the diagnostic accuracy of the PHQ-9 in primary care elderly

**DOI:** 10.1186/1471-2296-11-63

**Published:** 2010-09-01

**Authors:** Elizabeth Phelan, Barbara Williams, Kathryn Meeker, Katie Bonn, John Frederick, James LoGerfo, Mark Snowden

**Affiliations:** 1 Department of Medicine, Division of Gerontology and Geriatric Medicine, University of Washington, Seattle, WA, USA; 2 Department of Health Services, School of Public Health and Community Medicine, University of Washington, Seattle, WA, USA; 3 Department of Psychiatry and Behavioral Sciences, Division of Geriatric Psychiatry, University of Washington, Seattle, WA, USA; 4 Department of Medicine, Division of General Medicine, University of Washington, Seattle, WA, USA

## Abstract

**Background:**

The diagnostic accuracy of the Patient Health Questionnaire-9 (PHQ-9) for assessment of depression in elderly persons in primary care settings in the United States has not been previously addressed. Thus, the purpose of this study was to evaluate the test performance of the PHQ-9 for detecting major and minor depression in elderly patients in primary care.

**Methods:**

A prospective study of diagnostic accuracy was conducted in two primary care, university-based clinics in the Pacific Northwest of the United States. Seventy-one patients aged 65 years or older participated; all completed the PHQ-9 and the 15-item Geriatric Depression Scale (GDS) and underwent the Structured Clinical Interview for Depression (SCID). Sensitivity, specificity, area under the receiver operating characteristic (ROC) curve, and likelihood ratios (LRs) were calculated for the PHQ-9, the PHQ-2, and the 15-item GDS for major depression alone and the combination of major plus minor depression.

**Results:**

Two thirds of participants were female, with a mean age of 78 and two chronic health conditions. Twelve percent met SCID criteria for major depression and 13% minor depression. The PHQ-9 had an area under the curve (AUC) of 0.87 (95% confidence interval [CI], 0.74-1.00) for major depression, while the PHQ-2 and the 15-item GDS each had an AUC of 0.81 (95% CI for PHQ-2, 0.64-0.98, and for 15-item GDS, 0.70-0.91; *P *= 0.551). For major and minor depression combined, the AUC for the PHQ-9 was 0.85 (95% CI, 0.73-0.96), for the PHQ-2, 0.80 (95% CI, 0.68-0.93), and for the 15-item GDS, 0.71 (95% CI, 0.55-0.87; *P *= 0.187).

**Conclusions:**

Based on AUC values, the PHQ-9 performs comparably to the PHQ-2 and the 15-item GDS in identifying depression among primary care elderly.

## Background

The incidence of major depression in the general elderly population is approximately 15% per year and doubles after age 70 [1]. The prevalence of depression is higher in medical settings than in the community. Depression is associated with an increased risk of mortality [2], healthcare utilization [3], functional decline [4-6], and poorer quality of life [7,8]. Depression is however, quite responsive to treatment, and antidepressants are as effective for older adults as for younger individuals [1]. Care management has been shown to improve outcomes for elderly who are depressed and treated in primary care settings [9]. Thus, detection of depression among older adults in primary care is important, as it can be linked to effective treatment. Additionally, because most older adults seek care for their mental health issues in primary care [10], screening in primary care with referral to a mental health specialist for diagnostic evaluation of depressive symptoms is neither efficient nor practical in most instances.

To date, late life depression has been under-recognized and inadequately treated [11-13]. A fundamental challenge for the primary care provider is recognition of depression in the elder where depression symptoms and comorbid medical illness symptoms such as insomnia or anorexia overlap [14,15]. This situation is further complicated by frequent underreporting of depressive symptoms by older adults [12]. A brief screening tool that accurately identifies depression among elderly patients would make the identification of depression in primary care more straightforward, and improvements in identification and treatment of depression in elders might lead to improved function, survival, and quality of life. Thus, our study objective was to assess the diagnostic performance of the Patient Health Questionnaire-9 (PHQ-9) [16], a promising depression scale that has been validated with younger populations, and compare its performance to an established depression screening instrument, the 15-item Geriatric Depression Scale (GDS) [17], among elderly persons receiving health care in primary care settings. The PHQ-9 is the first self-report questionnaire designed for use in primary care that reflects the Diagnostic and Statistical Manual of Mental Disorders, fourth edition (DSM-IV) diagnostic criteria for depression, and so (through examining the pattern and number of items endorsed) can be used as a diagnostic tool for major and minor depression [16]. By contrast, other self-report instruments, including the GDS, do not map to the DSM depression diagnostic criteria and so cannot be used for depression diagnosis (only screening) [10], meaning that additional evaluation to establish a depression diagnosis must be conducted following a positive screen. While the PHQ-9 has been shown to be useful in general populations [16,18], including outpatient clinic settings in a variety of countries [19,20] and sensitive to change in the elderly [21], to our knowledge, its screening characteristics (sensitivity and specificity) had not been validated with elderly in a primary care setting in the United States. If a screening instrument's summary score (obtained through summing individual items scores) correlated strongly with gold-standard depression diagnosis obtained via in-person diagnostic interviews, this would be attractive, as it would make depression detection in primary care much more straightforward. A validation study examining this question is important, as the PHQ is being administered to adults of all ages, including the elderly, as part of national surveillance efforts to estimate the prevalence of mental health disorders in the United States [22]. We located only one study that focused specifically on the validity of the PHQ-9 in chronically ill elderly in primary care; this study was conducted in the Netherlands and focused on elders with diabetics and chronic obstructive pulmonary disease without known depression enrolled in a randomized trial of a nurse-led, psychological intervention [23]. We hypothesized that, because the PHQ-9 items map directly to DSM-IV criteria for depression, the PHQ-9 would have test performance characteristics at least comparable to the GDS but perhaps less ease of use (due to its more complex response format). We were interested in minor as well as major depression, because minor depression is more prevalent in primary care than major depression, is associated with adverse effects on functioning and may increase the risk of subsequent major depression in older primary care patients [24,25], increases health care use and costs [24], and may be responsive to treatment [26-28]. Because brief screening tools may be preferable for use in primary care settings, we also examined the sensitivity and specificity of the PHQ-2, an abbreviated version of the PHQ-9, in identifying patients with major and minor depression.

## Methods

### Setting

The study was conducted from November 2006 through August 2007 in two primary care clinics affiliated with the University of Washington, in Seattle, Washington, USA. These clinics were chosen because they provide primary care for elders and because clinic administrators and providers were supportive of the project.

### Study sample and procedures

Participants were consecutive established patients aged 65 or older presenting to the clinics for care. Those for whom the study procedures were not feasible due to severe dementia, unstable medical condition, or non-English fluency, were excluded. After informed consent was obtained, the PHQ-9 and 15-item GDS were administered to each participant by a research assistant. A geriatric psychiatrist or gerontologic psychiatric nurse practitioner trained in SCID administration, blinded to the results of the depression screening tests, conducted a diagnostic interview for depression, the Structured Clinical Interview for Depression (SCID). The SCID is considered the criterion standard for DSM-IV depression diagnosis in clinical research [29]. Information on demographic characteristics (age, gender, race) and chronic conditions was obtained from a questionnaire completed by the participant at the time of enrollment. All instruments were administered on the same day in the context of a routine clinic appointment. To avoid ordering effects, the order of administration of screening instruments and the SCID was varied in random fashion. The institutional review board at the University of Washington approved all procedures.

### Depression instruments

The PHQ-9 is a self-administered, nine-item questionnaire specific to depression that is available free to end users [16]. It was developed as a self-report version of the Primary Care Evaluation of Mental Disorders (PRIME-MD). It has several other features that make it attractive for use with older adults in primary care settings, including being substantially shorter than most other depression screening measures; having been originally developed and tested for use with medical patients, who are likely (as are elderly) to have high rates of physical symptoms consistent with either chronic medical illness or depression; having excellent test-retest reliability, excellent criterion [16] and construct validity [18], and responsiveness [21,30], or the capacity of an instrument to detect meaningful change over time [31].

Each of the nine items of the PHQ-9 is scored as 0 (not at all), 1 (several days), 2 (more than half the days), or 3 (nearly every day). As a screening tool, summing the 9 items, (score range 0-27 with 0 indicating no depressive symptoms and 27 indicating all symptoms occurring nearly daily), a score of ≥10 has been shown to have an 88% sensitivity and 88% specificity for major depression in a general medical population [18].

As a diagnostic tool, major depression is diagnosed if ≥5 of the 9 symptoms elicited have been present at least more than half the days in the past two weeks AND one of these symptoms is either depressed mood or anhedonia [18]. Minor depression is diagnosed if 2-4 symptoms have been present at least more than half the days in the past two weeks AND one of the symptoms is either depressed mood or anhedonia [18].

Depression severity can also be assessed with the PHQ-9 [16]. Kroenke et al suggested cutpoints to identify minimal (0-4), mild (5-9), moderate (10-14), moderately severe (15-19), and severe (≥20) depression [18]. These cutpoints have some empiric support, as demonstrated by a strong association between increasing PHQ-9 scores and worse scores on functional status (SF-20) measures, especially for scales most strongly correlated with depression [18].

The PHQ-2 is an abbreviated version (i.e., the first two items) of the PHQ-9 that inquires about depressed mood and anhedonia; it has been studied to a lesser extent than the PHQ-9 as a depression screening tool [32-34]. In studies of younger adults in primary care and obstetrics-gynecology settings, scores of ≥3 have a sensitivity of 83% and a specificity of 92% for major depression [33].

The 15-item GDS is the most commonly used, geriatric specific depression screen and thus represents the current "usual care" standard for geriatric depression screening [17]. Its brevity and dichotomized (yes, no) response format offer some ease of use advantages. In a study of persons 60 years or older from primary care practices, scores of >5 had a sensitivity of 92% and a specificity of 81% for major depression [17].

Published cutpoints for minor depression are not available for the three instruments for purposes of screening. However, the PHQ-9 can be used as a diagnostic tool for minor depression, since the items map directly to DSM minor depression criteria.

### Statistical analysis

Our analysis was conducted in three stages. First, descriptive statistics were calculated to characterize the sociodemographic and health characteristics of our sample. Next, sensitivity, specificity, and likelihood ratios (LRs) for detecting major depression for each of the three instruments (i.e., including the PHQ-2) were calculated over a range of cutpoints. Ninety-five percent confidence intervals (95% CI) were calculated for sensitivity and specificity using an online clinical calculator, available at http://statpages.org/ctab2x2.html. Third, receiver operating characteristic (ROC) analyses were conducted. ROC analyses combine instrument sensitivity and specificity into one measure (referred to as area under the curve, or AUC) for all possible cutpoints. AUC values range from ≤0.5 (no discriminatory ability) to 1.0 (perfect discrimination). An AUC of 0.84 implies that there is an 84% likelihood that a randomly selected person with depression will have a higher PHQ-9 score than a randomly selected non-depressed person. The AUC was measured to permit comparison of the diagnostic value of each instrument for detecting major depression, for the study group overall and by gender, ethnicity, age, and burden of comorbidity. A global non-parametric test for comparison of the 3 AUCs was calculated [35]. Lastly, we calculated sensitivity, specificity, LRs, and AUCs with depression broadened to include minor depression. Statistical analyses were performed using STATA v. 9.2 (Stata Corp., College Station, Texas).

## Results

### Participant flow

A total of 502 unique, established patients were seen in the clinics during the study period. Of these, 122 were not approached due either to being non-fluent in English (N = 64) or having severe dementia (N = 58). Of the 380 remaining, 227 met the age criterion and so were approached about the study by clinic staff. Of these, 121 were willing to speak with the research assistant about the study, and 71 agreed to participate.

### Baseline characteristics of participants

The 71 participants had a mean age of 78 years; nearly two-thirds were female, a third were non-white, and over three-quarters had a high school education (Table 1). They reported having two chronic medical conditions, on average, with hypertension, arthritis, and diabetes being the most common (reported by 63%, 49%, and 24%, respectively). About a quarter had a PHQ-9 score of 10 or greater, and one-fifth had a PHQ-2 score of three or greater. About half had a GDS score greater than five. The SCID was positive for major depression in 12% and for minor depression in 13%. Thirty percent needed help to complete the demographics questionnaire, 30% needed help to complete the GDS, and 37% needed help to complete the PHQ-9. Assistance was usually in the form of oral administration of the measures due to poor eyesight or difficulty using a pen. The yes/no response option format of the GDS was not clearly easier for participants to use than the four option response format of the PHQ-9. Participants took about five minutes to complete the PHQ-9, and about 25-50% longer to complete the GDS, on average.

**Table 1:** Demographic and health characteristics of participants at enrollment (N = 71)

Characteristic	
Age, years, mean ± SD*	78 ± 7
Female, %	62
Non-white, %	32
High school graduate, %	82
Chronic medical conditions	
Mean ± SD	2.3 ± 1.5
Median (interquartile range)	2 (2)
Three or more chronic medical conditions, %	42
Patient Health Questionnaire-9 (PHQ-9)	
Score, mean ± SD	5.9 ± 6.1
Score ≥10, %^†^	23 (16/71)
Patient Health Questionnaire-2 (PHQ-2)	
Score, mean ± SD	1.2 ± 1.6
Score ≥3, %^‡^	20 (14/71)
15-Item Geriatric Depression Scale (GDS)	
Score, mean ± SD	5.8 ± 1.8
Score >5, %^§^	48 (33/69)
Structured Clinical Interview for Depression (SCID)	
Major depression, %	12 (8/69)
Minor depression, %	13 (9/69)

* SD = standard deviation.

^† ^Scores of 10 or greater on the PHQ-9 suggest likely major depression in general medical populations [18].

^‡ ^Scores of 3 or greater on the PHQ-2 suggest possible depression in adults in primary care and obstetrics-gynecology settings [33].

^§ ^Scores greater than 5 on the 15-item GDS suggest major depression in older adults in primary care [17].

### Relative performance of depression screening instruments for major depression

The sensitivity and specificity of the screening measures were calculated using the SCID as the criterion standard for major and minor depression diagnosis. Using published, standard major depression cutpoints for these tests, seven of the eight (63%) majorly depressed participants were correctly classified with the PHQ-9 (cutpoint ≥ 10) and the PHQ-2 (cutpoint ≥ 3), while 100% were correctly classified with the GDS (cutpoint > 5) (Table 2). At these published cutpoints, the specificity was higher for the two PHQ measures (PHQ-9, 82%; PHQ-2, 85%) compared to the 15-item GDS, with a specificity of 58%. The LR positive represents how much the odds of having depression increase when a test is positive. At the standard cutpoints, the two PHQ measures had LR positives of about four, -- i.e., a positive screen is four times more likely to be seen in someone with major depression than in someone without -- while the 15-item GDS had a LR positive of 2.4, -- i.e., a positive screen is 2.4 times more likely to be seen in someone with major depression than in someone without the condition.

**Table 2:** Sensitivity and specificity of depression screening instruments for diagnosing major depression at various cutpoints*

Instrument and Cutpoint	Sensitivity (%) (95% CI)	Specificity (%) (95% CI)	+ Likelihood Ratio	- Likelihood Ratio
PHQ-9 Cutpoint				
≥8	88	75	3.6	0.16
	(56-98)	(71-77)		
**≥9**	**88**	**80**	4.4	0.16
	(56-98)	(76-82)		
*≥10*	*63*	*82*	3.5	0.46
	(33-86)	(78-85)		
≥11	63	84	3.8	0.45
	(33-85)	(80-87)		
≥12	63	84	3.8	0.45
	(33-85)	(80-87)		
				
PHQ-2 Cutpoint				
≥1	88	61	2.2	0.21
	(55-98)	(56-62)		
**≥2**	**75**	**67**	2.3	0.37
	(43-93)	(63-70)		
*≥3*	*63*	*85*	4.2	0.44
	(33-85)	(81-88)		
≥4	38	93	5.7	0.67
	(15-62)	(91-97)		
≥5	38	98	22.9	0.64
	(16-48)	(96-100)		
				
15-item GDS Cutpoint				
≥5	100	15	1.2	0.00
	(73-100)	(11-15)		
*≥6*	*100*	*58*	2.4	0.00
	(70-100)	(54-59)		
**≥7**	**75**	**77**	3.2	0.33
	(43-93)	(72-79)		
≥8	25	87	1.9	0.86
	(7-54)	(84-91)		
≥9	13	93	1.9	0.94
	(2-37)	(92-97)		

* Cutpoints are specific sum scores that distinguish between individuals with and without the disorder. Bolded cutpoints indicate the optimum balance between sensitivity and specificity, while italicized cutpoints are those that are typically cited in the literature as being those that optimize sensitivity and specificity for the detection of major depression for each respective instrument when applied to the general population.

^† ^CI = confidence interval.

Table 3 reports results from the ROC analysis, which gives a global assessment of the discriminatory power of each instrument. Overall, the AUC for the PHQ-9 was comparable to the AUC for the PHQ-2 and the 15-item GDS (0.87 for PHQ-9 vs. 0.81 for both the PHQ-2 and the 15-item GDS, *P *= 0.551).

**Table 3:** Receiver operating curve analyses for major depression for each screening instrument

	N*	PHQ-9 AUC^†^ (95% CI)^‡^	PHQ-2 AUC (95% CI)	GDS AUC (95% CI)
Overall	69	0.87 (0.74-1.00)	0.81 (0.65-0.98)	0.81 (0.70-0.91)
				
Gender				
Male	26	0.88 (0.72-1.00)	0.91 (0.78-1.00)	0.76 (0.58-0.93)
Female	43	0.85 (0.61-1.00)	0.70 (0.37-1.00)	0.84 (0.70-0.98)
				
Race				
White	47	0.88 (0.73-1.00)	0.87 (0.75-0.99)	0.85 (0.74-0.95)
Non-white^‡^	22	0.93	0.31	0.90
				
Age				
< 80 years	38	0.92 (0.81-1.00)	0.81 (0.54-1.00)	0.82 (0.68-0.96)
≥80 years	31	0.80 (0.52-1.00)	0.83 (0.64-1.00)	0.80 (0.62-0.98)
				
Comorbidities				
< 3	41	0.93 (0.82-1.00)	0.92 (0.85-1.00)	0.79 (0.55-1.00)
≥3	28	0.80 (0.57-1.00)	0.71 (0.43-0.99)	0.82 (0.67-0.97)

* N is for PHQ-9 and PHQ-2; GDS is missing for one participant.

^† ^AUC = area under the receiver operating characteristic curve. Values range from ≤0.5 (no discriminatory ability) to 1.0 (perfect discrimination -- in this case, of depressed from non-depressed).

^‡ ^CI = confidence interval.

^§ ^Only one of the 22 non-white participants had a positive SCID, and therefore no confidence interval could be calculated.

As shown in Table 3, the AUC for the PHQ-9 and 15-item GDS was similar for men and women, whereas for the PHQ-2, the AUC was lower for women. This same pattern of AUCs for the three instruments held for white and non-white participants. The PHQ-9 appeared somewhat more discriminatory for those under the age of 80 years, whereas the PHQ-2 and the 15-item GDS discriminated comparably for these subgroups. The PHQ-9 and PHQ-2 AUC values were highest for participants with less than three comorbidities (0.93 and 0.92, respectively), whereas the AUC for the 15-item GDS was similar regardless of comorbidity burden.

### Relative performance of depression screening instruments using broadened definition of depression

When the broadened definition of depression was used (i.e., including minor and major depression), the sensitivity of all three measures worsened as compared with their case detection for major depression alone (Table 4). For example, at the PHQ-9 cutpoint of ≥10, the sensitivity using the broadened definition was 59% as opposed to 63%; at the PHQ-2 cutpoint of ≥3, the sensitivity was 53% as opposed to 63%. For the 15-item GDS, at the cutpoint of >5, the sensitivity was 81% as opposed to 100%. AUC values using the broadened definition of depression were: PHQ-9 = 0.85 (95% CI, 0.73-0.96); PHQ-2 = 0.80 (95% CI, 0.68-0.93); and 15-item GDS = 0.71 (95% CI, 0.55-0.87); *P *= 0.187 for comparison of AUCs for the three instruments.

**Table 4:** Sensitivity and specificity of each screening instrument using a broadened definition of depression*

Instrument and Cutpoint	Sensitivity (%) (95% CI)	Specificity (%) (95% CI)	+ Likelihood Ratio	- Likelihood Ratio
PHQ-9 Cutpoint				
≥6	77	69	2.5	0.34
	(56-90)	(63-74)		
≥7	77	77	3.3	0.31
	(56-90)	(70-81)		
**≥8**	**77**	**83**	4.4	0.28
	(57-89)	(76-87)		
≥9	71	87	5.2	0.34
	(51-85)	(80-91)		
≥10	59	89	5.1	0.47
	(40-74)	(82-93)		
				
PHQ-2 Cutpoint				
**≥1**	**82**	**67**	2.5	0.26
	(62-94)	(61-71)		
≥2	71	73	2.6	0.40
	(50-86)	(66-78)		
≥3	53	90	5.5	0.52
	(35-67)	(85-95)		
≥4	35	98	18.4	0.66
	(21-40)	(94-100)		
≥5	18	98	9.2	0.84
	(7-22)	(95-100)		
				
15-item GDS Cutpoint				
≥4	94	4	0.98	1.63
	(85-99)	(1-5)		
≥5	88	14	1.0	0.93
	(71-96)	(8-16)		
**≥6**	**81**	**62**	2.1	0.30
	(60-93)	(55-65)		
≥7	56	79	2.7	0.55
	(36-74)	(73-84)		
≥8	31	90	3.3	0.76
	(16-46)	(86-95)		

* Bolded cutpoints indicate the optimum balance between sensitivity and specificity.

^‡ ^CI = confidence interval.

## Discussion

This study demonstrated that, used as a screening instrument, the PHQ-9 performed comparably to the PHQ-2 and the 15-item GDS for the purposes of major depression detection in elderly individuals in primary care. Broadening the definition of depression to include minor along with major depression did not improve performance of any of the three screening instruments. Self-administration of the PHQ-9 did not result in substantially greater need for assistance, compared to the GDS, among our study sample. The PHQ-9 performed comparably regardless of gender or race and somewhat better for younger elders and for those with less chronic illness. The prevalence of major depression in our sample was 12%, comparable to that found in other studies of elderly across a range of health care settings [36], and the prevalence of minor depression was 13%.

This study was motivated by a desire to address the challenge of depression recognition by non-psychiatric physicians, and in particular, recognition of depression in the elderly where depression symptoms and symptoms of comorbid medical illness overlap [13-15]. The utility of the PHQ-9 for detection of depression in other populations has been previously documented [16,18,37-46]; however, to our knowledge, ours is the first study to examine its screening characteristics for both major and any (major or minor) depression with an exclusively older adult study sample in a primary care clinic setting in the United States. With an AUC for major depression in our study of 0.87, the performance of the PHQ-9 in identifying major depression was somewhat worse than has been demonstrated for general medical populations, where the AUC was 0.95 [18]. In the only other published study that we were able to locate that focused specifically on the validity of the PHQ-9 in an elderly sample, the AUC for major depression was 0.92 [23]. It is of interest that, despite substantial differences between our two study samples (i.e., northern European vs. United States origin; enrolled in a randomized controlled trial vs. consecutively enrolled from a primary care clinic; excluded if carried a prior diagnosis of depression or other psychiatric disorder vs. depression and/or other psychiatric disorder other than severe dementia did not preclude participation) our results for major depression detection through use of the PHQ-9 are quite comparable. Taken together, they provide evidence in support of the instrument's validity as a screening instrument for elders with chronic illness, including those who carry a depression diagnosis or other psychiatric disorder.

Of note, the low specificity (i.e., 58%) of the 15-item GDS for major depression at the standard cutpoint (i.e., >5) may limit its use with older persons similar to those in our study, because many would need follow-up evaluation to receive a specific depression diagnosis. By contrast, both the PHQ-9 and PHQ-2 have better sensitivity and specificity at the standard major depression cutpoints; however, compared to the 15-item GDS, both risk missing cases because of their lower sensitivity at those cutpoints.

It is of interest that the optimal cutpoints for detection of major depression for the instruments evaluated in this study varied from previously published cutpoints for general medical populations. Specifically, a PHQ-9 cutpoint of ≥9, a PHQ-2 cutpoint of ≥2, and a GDS cutpoint of ≥7 offered the best combination of sensitivity and specificity for our study participants. The need to modify cutpoints to achieve the best balance between sensitivity and specificity for elderly populations has been observed in other studies [23,36]. More research in this area appears warranted.

Even with modification of cutpoints, our results demonstrate that the PHQ-2 performs less well for detection of major depression among primary care elderly as compared to younger adults, where sensitivity and specificity have been reported to be 83% and 92% respectively [33]. Our finding is, however, consistent with the one study that examined the utility of the PHQ-2 for detecting major depression in elderly primary care patients and found a sensitivity of only 79% and specificity of 58% with a cutpoint of 1 or greater [36].

In contrast to other studies, wherein broadening the definition of depression to include depression of lesser severity and dysthymia improved the sensitivity of depression screening instruments [23,36], our study demonstrated that sensitivity was not improved through this maneuver. Sensitivity in our study was determined to have decreased as the result of an increase in false negatives that occurred when the definition of depression was broadened (data not shown). However, AUC values derived using our broadened definition remained comparable to those derived using the narrower (major depression only) definition and still in the acceptable range of discrimination.

This study was limited by several factors. First, we did not assess the percentage of patients already diagnosed with depression or the percentage on antidepressants. Second, our sample was in essence a convenience sample, rather than a nationally representative sample of elders receiving care in primary care settings. Additionally, our sample size was smaller than anticipated, due in part to substantial numbers of clinic patients being ineligible to participate because of language barriers (non-fluent in English) and cognitive dysfunction, and in part to a relatively low acceptance of study participation. The small sample size likely contributed to the small number (i.e., 8) of cases of major depression in the study, and this small number in turn limits interpretation of the statistical comparisons of the screening instruments. The low study participation may have affected the prevalence of depression in our study sample, as one could imagine that those with depression might be either more or less likely to agree to participate in a study of depression screening. Lastly, the validity of the depression instruments for identifying depression in persons with dementia or those who are non-English speaking could not be ascertained.

These limitations notwithstanding, this study has several strengths. First, our study was conducted with a diverse elderly sample, and we were able to describe the extent and nature of chronic medical conditions of study participants. Secondly, the depression screening instruments were mostly self-administered, with assistance as needed from the research assistant, while the SCID was conducted by the study psychiatrist/study nurse practitioner, thus minimizing the likelihood of agreement of the screening instruments with the criterion standard. Thirdly, the psychiatrist and nurse practitioner who administered the SCID were blinded to the results of the depression screening instruments and to any treatment that the study participant may have already been receiving for depression.

## Conclusions

In balance, these data suggest that the PHQ-9 performs comparably to the 15-item GDS when used as a screening instrument for detection of depression among elderly persons in primary care settings. Because of its brevity and its utility in making specific, DSM-IV based, depression diagnoses, the PHQ-9 represents a reasonable alternative to the GDS, particularly in situations where referral to a mental health provider for definitive diagnostic evaluation is neither an option nor desired by the patient.

## Competing interests

The authors declare that they have no competing interests.

## Authors' contributions

EAP conceptualized the design of the study, obtained funding for the project, worked closely with administrators and staff of participating clinics to ensure acceptance and understanding of the clinic's role in study procedures, obtained IRB approval for all study procedures, interpreted data, and had primary responsibility for preparation of the manuscript. BW performed data analyses and assisted with interpretation of the data and drafting of the manuscript. KM served as the research assistant. She recruited all study participants and ensured that they completed all study procedures. She was responsible for data tracking and management. KJB and JTF conducted diagnostic interviews for depression. JPL contributed to interpretation of the data, facilitated clinic involvement, and provided critical review of the manuscript. MS contributed to study conception and design, provided content expertise and logistical support for the project, interpreted data, and provided critical review and final approval of the manuscript. All authors approved the final manuscript.

## Authors' information

Dr. Snowden is a practicing geriatric psychiatrist and director of the Geriatric Psychiatry Services Program at Harborview Medical Center, Seattle, Washington. He chaired an expert panel that conducted a systematic literature review of depression screening instruments for community-based (e.g., senior centers, adult day health, senior housing) settings.

## Pre-publication history

The pre-publication history for this paper can be accessed here:

http://www.biomedcentral.com/1471-2296/11/63/prepub
